# Phosphodiesterase 8B gene polymorphism in women with recurrent miscarriage: A retrospective case control study

**DOI:** 10.1186/1471-2350-13-121

**Published:** 2012-12-13

**Authors:** Michaela Granfors, Helena Karypidis, Frida Hosseini, Lottie Skjöldebrand-Sparre, Anneli Stavreus-Evers, Katarina Bremme, Britth-Marie Landgren, Inger Sundström-Poromaa, Anna-Karin Wikström, Helena Åkerud

**Affiliations:** 1 Department of Women’s and Children’s Health, Uppsala University, Uppsala, Sweden; 2 Department of Clinical Sciences, Division of Obstetrics and Gynaecology, Karolinska Institutet and Danderyd Hospital, Stockholm, Sweden; 3 Department of Women’s and Children’s Health, Karolinska Institutet, Stockholm, Sweden; 4 Department of Clinical Sciences, Intervention and Technology, Karolinska Institutet, Karolinska University Hospital Huddinge, Stockholm, Sweden

**Keywords:** Phosphodiesterase 8B, Recurrent miscarriage, Single nucleotide polymorphism, Thyroid

## Abstract

**Background:**

Recurrent miscarriage affects approximately 1% of all couples. There is a known relation between hypothyroidism and recurrent miscarriage. Phosphodiesterase 8B (PDE8B) is a regulator of cyclic adenosine monophosphate (cAMP) with important influence on human thyroid metabolism. Single nucleotide polymorphism (SNP) rs 4704397 in the PDE8B gene has been shown to be associated with variations in serum Thyroid Stimulating Hormone (TSH) and thyroxine (T4) levels. The aim of this study was to investigate whether there is an association between the SNP rs 4704397 in the PDE8B gene and recurrent miscarriage.

**Methods:**

The study was designed as a retrospective case control study. 188 cases with recurrent miscarriage were included and compared with 391 controls who had delivered at least once and with no history of miscarriage or assisted reproduction.

**Results:**

No difference between cases and controls concerning age was found. Bivariate associations between homozygous A/A (OR 1.57, 95% CI 0.98-2.52) as well as G/G carriers (OR 1.52, 95% CI 1.02-2.25) of SNP rs 4704397 in PDE8B and recurrent miscarriage were verified (test for trend across all 3 genotypes, p = 0.059). After adjustment for known confounders such as age, BMI and smoking the association between homozygous A/A (AOR 1.63, 95% CI 1.01 - 2.64, p = 0.045) and G/G (AOR 1.52, 95% CI 1.02 - 2.27, p = 0.039) carriers of SNP rs 4704397 in PDE8B and recurrent miscarriage remained.

**Conclusions:**

Our findings suggest that there is an association between homozygous A/A as well as homozygous G/G carriers of SNP rs 4704397 in PDE8B and recurrent miscarriage.

## Background

Recurrent miscarriage affects approximately 1% of all couples trying to conceive and is defined as three or more consecutive pregnancy losses before 20 weeks of amenorrhoea [1,2]. Predisposing factors include maternal age, parental chromosomal aberrations, uterine abnormalities, antiphospholipid syndrome, immunological and thrombophilic disorders, and endocrine diseases such as hypothyroidism and diabetes mellitus [1]. Unlike sporadic spontaneous miscarriage, recurrent miscarriage more often occurs despite normal fetal cytogenetic findings, and in 50% of cases the underlying cause remains unexplained [1,3-5]. Hypothyroidism is a common disease, with an estimated incidence of 0.3% to 0.5% for overt and 3% for subclinical hypothyroidism in pregnant women [1,6-8]. There is a known relation between hypothyroidism and recurrent miscarriage [1].

The human phosphodiesterase type 8B (PDE8B) gene is located at human chromosome 5q14.1 in intron 1 and encodes a high affinity cyclic adenosine monophosphate (cAMP) specific nucleotide phosphodiesterase [9-11]. The PDE8B gene is abundantly expressed in the thyroid but has also been detected in human placenta and ovaries [9,10,12,13]. Based on a recent genome-wide association study, six different single nucleotide polymorphisms (SNP) in the PDE8B gene were associated with increased serum concentrations of thyroid stimulating hormone (TSH) [14]. The strongest association with increased TSH levels (although in the normal range) was reported for one specific SNP in PDE8B, rs 4704397, which is found in the promoter region of the gene [14,15]. A recent meta-analysis confirmed the association between SNP rs 4704397 in PDE8B and elevated TSH levels, but also found an association with decreased levels of free T4, although free T3 levels were normal [16]. In addition, the association between SNP rs 4704397 in PDE8B and levels of TSH has been confirmed in pregnant women, although no association with free T3 or T4 levels was found [17].

In SNP rs 4704397 of PDE8B an adenine (A) nucleotide is replaced by a guanine (G). The association between the polymorphism and high levels of TSH and low free T4 levels, indicating relative hypothyroidism, is found in homozygous carriers of A/A [16]. Based on previous results it has been proposed that the SNP rs 4704397 in PDE8B and in particular the presence of A alleles might induce increased phosphodiesterase activity in PDE8B, thereby reducing the ability of the thyroid gland to generate free T4 when stimulated by TSH [14]. Related to this, we hypothesized that homozygous carriers of A/A would be at increased risk of recurrent miscarriage. The aim of this study was to investigate whether there is an association between the SNP rs 4704397 in the PDE8B gene and recurrent miscarriage.

## Methods

### Study population

The study was designed as a case–control study. Cases (n = 188) were recruited from the Department of Obstetrics and Gynaecology at Uppsala University Hospital, Karolinska University Hospital, Huddinge University Hospital and Danderyd University Hospital, Sweden. Eligible cases with a diagnosis of recurrent miscarriage, defined as three or more verified consecutive miscarriages in the first or second trimester of pregnancy (5–21 completed weeks of gestation), were identified in the out-patient registers of the participating clinics and invited to participate in the study. The women were included between April 29 2009 and June 30 2010. Women with known risk factors for recurrent miscarriage, such as systemic lupus erythematosus, diabetes mellitus type 1, severe thrombophilia and major chromosomal aberrations were not included in the study. Furthermore, two women with hyperthyroidism were excluded.

The control subjects (n=391) were matched for age at first planned pregnancy and were randomly chosen from the Uppsala University Hospital biobank of pregnant women. Since May 31 2007, all women aged 18 and older attending the second trimester (16–19 weeks of gestation) routine ultrasound scan at Uppsala University Hospital have been approached for inclusion in this biobank, and inclusion to our study was ongoing until June 30 2010. In the control group, none had a history of miscarriage and 74.9% had at least two spontaneous pregnancies, including the ongoing pregnancy, resulting in a term (≥37 weeks) birth of a live infant. Beyond that, the same inclusion and exclusion criteria were applied for the control group as for the cases.

Both cases and controls attended a brief health examination including measurements of weight and height and answered standardised questions on reproductive history. Further, we reviewed the medical records to obtain relevant information on pregnancy outcomes, health problems and medication.

According to routine clinical procedures, TSH-levels were analysed in all women when diagnosed with recurrent miscarriage. In the case-group, hypothyroidism was defined as TSH above the current defined upper limit of the reference range at the different hospitals. Pregnant controls at high risk for thyroid disease were subjected to selected TSH screening according to local guidelines based on international recommendations [6]. This case-finding procedure was applied in first or early second trimester of pregnancy, before routine ultrasound scan and inclusion in the study.

The study was approved by the Regional Ethics Committee of the Medical Faculty of Uppsala University Hospital and the Ethical Committee of the Karolinska Institutet, Stockholm. Informed consent was obtained from all women included in the study.

### Blood sample collection

Blood samples were collected in EDTA-containing tubes and centrifuged at 1500 g for 10 min. Plasma and buffy coat were separated and stored at −20°C.

### SNP-analysis

Genomic DNA was extracted from blood using QIAamp^®^ DNA Blood Maxi kits (Qiagen, Venlo, the Netherlands). The samples were genotyped for the SNP rs4704397 of PDE8B, using the TaqMan^®^ SNP Genotyping Assay (Applied Biosystems, Foster City, CA, USA). Briefly, PCR reactions were performed in a 96 well plate in a total volume of 25 μl for each reaction. Each reaction consisted of 1x TaqMan Universal PCR Master Mix (PCR buffer, ROX passive reference dye, dNTPs and AmpliTaq Gold polymerase), 1x SNP Genotyping Assay (sequence-specific forward and reverse primers to amplify the polymorphic sequence of interest, i.e. HRG exon 5, TaqMan^®^ MGB probes labelled with VIC ^®^dye to detect allele 1 sequence and with FAM™ to detect allele 2 sequence) and 10 ng of genomic DNA. Cycling conditions were initiated for 10 min at 95°C, followed by 40 cycles of 15s at 92°C and 1 minute at 60°C. Real time fluorescence detection was performed. The Sequence Detection System (SDS) Software (Applied Biosystems) was used to plot fluorescence (Rn) values based on the signals from each well. The plotted fluorescence signals indicated which alleles were present in each sample.

### Statistics

Demographic and clinical characteristics were compared between cases and controls or genotype groups using t-test and Chi-square tests. ANOVA was used for continous variables. Adjusted odds ratios (AOR) for recurrent miscarriage were calculated in multivariate regression analyses using the following variables: maternal age as completed years at the first pregnancy or first miscarriage, body mass index (BMI, kg/m^2^) defined as BMI recorded at inclusion (cases) or BMI at first antenatal visit (controls), smoking during pregnancy (yes/no) defined as smoker during ≥ 1 pregnancy ending with miscarriage (cases) or smoker at first visit to the prenatal centre in gestational week 10 (controls), and genotype (A/G as reference). Only variables with possible association with each outcome (p < 0.25) in the bivariate analyses were entered into the final model. All significance tests were two-tailed. P-values < 0.05 were considered as statistically significant. All statistical analyses were performed using SPSS 16.0 for Windows software pack (SPSS, Chicago, IL).

## Results

### Background characteristics and genotyping of PDE8B

Demographic data and clinical characteristics for cases and controls are shown in Table 1. There were no differences between cases and controls concerning age (p = 0.99). As expected, BMI was higher (p=0.033) and smoking (18.7% vs. 10.7%, p =0.015) and hypothyroidism (8.5% vs. 3.1%, p = 0.006) were more common in women with recurrent miscarriage.

**Table 1:** Demographic data and clinical characteristics of the study population

	**Cases (n = 188)**	**Controls (n = 391)**	**p-value**
Age, years	30.1 ± 5.8	30.1 ± 5.8	0.989
BMI, kg/m^2^	24.7 ± 4.8	23.9 ± 4.0	0.031
Smokers, n (%)	34 (18.1%)	42 (10.7%)	0.015
Hypothyroidism, n (%)	16 (8.5%)	12 (3.1%)	0.006

Values are mean ± standard deviation; n, numbers of women; BMI, body mass index: BMI at inclusion (cases), BMI at first antenatal visit (controls); Smokers: smoker during ≥1 pregnancy ending with miscarriage (cases), smoker at first visit to the prenatal center in gestational week 10 (controls); Hypothyroidism: elevated serum TSH-levels according to local laboratory limits.

Among the 579 genotyped women, 110 (19.0%) were homozygous carriers of A/A, 266 (45.9%) were heterozygous carriers (A/G) and 203 (35.1%) were homozygous carriers of G/G (Table 2a). Homozygous A/A and G/G carriers, compared to heterozygous carriers, were more common among women with recurrent miscarriage (Table 2b). Homozygous and heterozygous carriers were in accordance with the Hardy Weinberg equilibrium.

**Table 2:** Demographic data and clinical characteristics of the study population according to the SNP rs 4704397 genotype

	**A/A**	**A/G**	**G/G**
	**(n = 110)**	**(n = 266)**	**(n = 203)**
Age, years	29.9 ± 5.1	30.5 ± 6.0	29.6 ± 5.8
BMI at first antenatal visit, kg/m^2^	23.6 ± 4.2	24.5 ± 4.4	24.1 ± 4.3
Smokers, n (%)	16 (14.5%)	31 (11.7%)	29 (14.3%)

Values are mean ± standard deviation. No significant differences between the groups exist.

### Genotype and risk of recurrent miscarriage

A bivariate analysis was performed including all known confounders. Based on the finding (Table 2b) that homozygous A/A and G/G carriers seemed to be more common in women with recurrent miscarriage, heterozygous A/G carriers were chosen as reference group in the logistic regression analyses. Bivariate associations between homozygous A/A as well as G/G carriers of SNP rs 4704397 in PDE8B and recurrent miscarriage were verified and a test for trend across all 3 genotypes was performed (Table 4). After adjustment for known confounders such as age, BMI and smoking, the association between homozygous A/A (AOR 1.63, 95% CI 1.01 - 2.64, p = 0.045) and G/G (AOR 1.52, 95% CI 1.02 - 2.27, p = 0.039) carriers of SNP rs 4704397 in PDE8B and recurrent miscarriage remained.

**Table 2:** Distribution of SNP rs 4704397 genotype among controls and cases

	**Controls (n=391)**	**Recurrent miscarriage (cases, n=188)**	
		All	Hypothyroidism
A/A	69 (17.6%)	41 (21.8%)	3 (18.8%)
A/G	193 (49.4%)	73 (38.8%)	4 (25.0%)
G/G	129 (33.0%)	74 (39.4%)	9 (56.2%)

**Table 3:** Factors associated with recurrent miscarriage

		**Unadjusted OR (95 % CI)**	**Adjusted OR**^**1**^**(95 % CI)**	**p-value (Adjusted OR)**
Age	years	1.0 (0.97-1.03)	0.99 (0.96-1.02)	0.500
BMI	kg/m^2^	0.96 (0.92 – 1.00)	0.96 (0.92-0.96)	0.026
Smoker	No	1	1	
	Yes	1.84 (1.12 - 3.00)	1.86 (1.12 – 3.09)	0.017
SNP rs4704397	A/G*	1	1	
	A/A	1.57 (0.98 - 2.52)	1.63 (1.01 - 2.64)	0.045
	G/G	1.52 (1.02 - 2.25)	1.52 (1.02 - 2.27)	0.039

^1^adjusted for age, smoking, BMI and genotype.

*Test for trend across genotypes in relation to recurrent miscarriage was p= 0.059.

## Discussion

PDE8B is known to be abundantly expressed in the thyroid and is of importance for thyroid function [9,12,13,15]. Increased levels of TSH have previously been shown in homozygous A/A carriers of SNP rs 4704397 in PDE8B and we hypothesized that there might be an association between A allele carriers and recurrent miscarriage. In our study, associations between homozygous A/A as well as homozygous G/G carriers of SNP rs 4704397 in PDE8B and recurrent miscarriage were found.

It has been shown that deranged levels of TSH in serum are related to inheritable factors in 65% of cases [18,19]. According to a genome-wide association scan in a Sardinian population, the specific SNP rs 4704397 in PDE8B has a strong association with increased levels of TSH and explains 2.3% of the variance in TSH. Each copy of the A allele was associated with an average increase of 0.13 μIU/ml in TSH serum concentrations [14].

Limitingly, as the study population was evaluated at clinics with different reference ranges for TSH analyses, and serum was not stored at time of diagnosis, TSH levels were unavailable for further analysis. Thus, we were not able to draw conclusions about whether the increased risk of recurrent miscarriage in homozygous A/A carriers in our material was mediated through relatively increased TSH levels. However, the prevalence of hypothyroidism was slightly, but not enough to be statistically significant, greater in homozygous A/A than in G allele carriers.

Based on the fact that homozygous A/A and G/G carriers, compared to heterozygous, were more common among women with recurrent miscarriage, we decided to use heterozygous carriers as references. This concept has been used before in other studies. When several different folate-metabolizing genes were analyzed, heterozygous rather than wild-type homozygous carriers, appeared to have higher pregnancy success rate after IVF treatment [20,21].

Not only A/A, but also homozygous carriers of the G/G SNP rs 4704397 in PDE8B were more common among women with recurrent miscarriage. This finding is novel and the mediator of the association is unknown. The association is presumably not mediated by inadequate thyroid metabolism, since no association between homozygous G/G carriers and thyroid disease has been identified in previous studies. Consistent with this, the prevalence of hypothyroidism in G/G carriers did not differ from that of A/G carriers in our study population (Table 2a). It is very likely that another mediator is underlying this association.

Moreover, it is noteworthy that G/G carriers were more common among hypothyroid women with recurrent miscarriage, compared to both controls and all cases (Table 2b). Thus, concerning recurrent miscarriage, it seems to be a disadvantageous combination to be G/G carrier and to suffer from hypothyroidism. However, it is important to remember that the number of women with hypothyroidism is quite small in this material.

The importance of PDE8B in the placenta and human ovaries is unknown, but the relevance of phoshodiesterases (PDE) in human reproduction has been discussed [10,13]. The mechanisms that regulate oocyte maturation *in vivo* and *in vitro* are still not well understood but the second messenger, cyclic adenosine mono-phosphate (cAMP), plays a critical role in maintaining the oocytes at meiotic arrest in the diplotene stage of the first meiotic prophase [22]. The PDEs inactivate and degrade cAMP in oocytes as a response to the ovulatory luteinizing hormone pulse and have thus been proposed as factors of importance for regulating oocyte maturation [23-25]. Furthermore, it has been shown that levels of cAMP in the oocyte at meiotic resumption correlate with oocyte competence and embryonic development [26,27]. Based on the knowledge that PDE activity is relevant for levels of cAMP in oocytes, the importance of SNP rs 4704397 in PDE8B for regulation of oocyte maturation would be one pathway of interest for further study.

Another limitation with our study is that cases and controls were screened for thyroid disease in different ways. According to routine clinical procedures, TSH-levels in all women with recurrent miscarriage were analyzed. Pregnant controls were subjected to selected TSH screening when considered to be at high risk for thyroid disease, in accordance with international guidelines [6]. Thus, the frequency of hypothyroidism might have been underestimated in the control group, which is of importance when the results are evaluated.

Of course, we cannot exclude our results concerning SNP associations to be false positive. Generally, there is a high false positive rate in small genetic association studies due to low prior probability [28]. However, SNP rs 4704397 in PDE8B has been shown to be associated with variations in serum TSH levels in several studies [14-17], and there is a well known association between hypothyroidism and recurrent miscarriage [1]. To our knowledge, this is the first study examining a possible association between SNP rs 4704397 in PDE8B and recurrent miscarriage. To confirm our findings, it would be important to increase the sample size and replicate in an independent cohort in a further study.

## Conclusions

Our findings suggest that there is an association between homozygous A/A as well as homozygous G/G carriers of SNP rs 4704397 in PDE8B and recurrent miscarriage.

In our study, different alleles of SNP rs 4704397 in PED8B were associated with the same outcome, recurrent miscarriage, but the explanation for this association is a subject for speculation. Genetic variability plays a role in many different diseases and has been proposed to be of relevance in screening for distinct syndromes with known genetic defects, and it might also be used for prediction of success related to disease treatment [29,30].

Hypothetically, this specific SNP may be used for individual counselling and for optimising treatment to prevent recurrent miscarriage, based on which allele the woman is a carrier of.

However, larger studies with independent replication are needed to confirm our results and their clinical importance.

## Competing interests

The authors declare no conflict of interest.

## Authors’ contributions

HK, FH, LSS, ASE, KB BML and ISP designed the study and were responsible for data collection. HÅ designed the study, analyzed the data and drafted the manuscript. MG and AKW analyzed the data and drafted the manuscript. All authors read and approved the final manuscript.

## Pre-publication history

The pre-publication history for this paper can be accessed here:

http://www.biomedcentral.com/1471-2350/13/121/prepub
